# Epigallocatechin gallate inhibits hepatitis B virus infection in human liver chimeric mice

**DOI:** 10.1186/s12906-018-2316-4

**Published:** 2018-09-06

**Authors:** Yu-Heng Lai, Cheng-Pu Sun, Hsiu-Chen Huang, Jui-Chieh Chen, Hui-Kang Liu, Cheng Huang

**Affiliations:** 1 Department of Chemistry, Chinese Culture University, Taipei, 11114 Taiwan; 2 Institute of Biomedical Sciences, Academia Sinica, Taipei, 11529 Taiwan; 3 Department of Applied Science, National Tsing Hua University South Campus, Hsinchu, 30014 Taiwan; 4 Department of Biochemical Science and Technology, National Chiayi University, Chiayi, 60004 Taiwan; 5 National Research Institute of Chinese Medicine, Ministry of Health and Welfare, Taipei, 11221 Taiwan; 6 Program in Clinical Drug Development of Chinese Herbal Medicine, Taipei Medical University, Taipei, 11001 Taiwan; 7 Department of Biotechnology and Laboratory Science in Medicine, National Yang-Ming University, No. 155, Sec. 2, Linong St., Beitou District, Taipei, 11221 Taiwan; 8 Department of Earth and Life Sciences, University of Taipei, Taipei, 11153 Taiwan

**Keywords:** Hepatitis B virus, EGCG, HBsAg, Human liver chimeric mice

## Abstract

**Background:**

Persistent hepatitis B virus (HBV) infection causes liver cirrhosis and hepatocellular carcinoma and constitutes a major worldwide health problem. Currently, anti-HBV drugs are limited to peginterferon and nucleos(t)ide analogs, which are costly and have considerable side effects; the development of novel, effective anti-HBV agents is crucial.

**Methods:**

Catechins are a major group of compounds found in green tea extract and epigallocatechin gallate (EGCG) has been shown to have antiviral properties, including inhibition of cellular entry by HBV. FRG (Fah^−/−^/ Rag2^−/−^/ IL-2Rγ^/−^) mice were used in this study to generate chimeras carrying human primary hepatocytes, to facilitate investigation of the inhibitory effect of EGCG on HBV infection.

**Results:**

Here, we show the inhibitory effect of EGCG on HBV infection and replication in HuS-E/2 cells. The inhibitory effect of EGCG on HBV infection in vivo was confirmed by monitoring HBV DNA and HBsAg in serum and immunostaining the liver tissues of the human liver chimeric mice.

**Conclusions:**

The effects of EGCG suggest a robust strategy for the treatment of HBV infection and EGCG may have therapeutic potential for the treatment of HBV-associated liver diseases.

## Background

Hepatitis B virus (HBV) infection is a major cause of acute and chronic viral hepatitis in humans, with the risk of development of cirrhosis and hepatocellular carcinoma (HCC) [1]. Today, approximately 350 million individuals are chronically infected, despite of the availability of an effective vaccine for more than 25 years [2]. There is an approximately 100-fold greater relative risk of HCC among HBV carriers than non-carriers [2]. However, many HBV-infected patients have not been treated with antiviral drugs, including interferon-alpha and nucleotide analogues that inhibit the viral reverse transcriptase, because of the adverse side effects and development of drug resistance, as well as the high cost of treatment [3]. Therefore, it is crucial to develop safe and effective, as well as affordable, anti-HBV agents that inhibit viral replication and improve the clinical outcome of HBV patients.

HBV is a small DNA virus with a nucleocapsid that protects the 3.2 kb genome [4]. The nucleocapsid is surrounded by an envelope, which contains three types of hepatitis B surface antigen (HBsAg), the small (S), medium (M) and large (L) forms, with distinct functions [5]. These proteins are encoded by one open reading frame, with three in-phase start codons. The M form of HBsAg (MHBsAg) has a hydrophilic, 55 amino acids (aa) N-terminal extension of the S domain, known as the pre-S2 domain [6]. The L form of HBsAg (LHBsAg) contains the pre-S1 domain, which extends from the pre-S2 domain for another 108 or 119 aa, dependent on the genotype [6]. The LHBsAg protein plays pivotal roles in the viral entry process [7, 8] and, recently, sodium taurocholate cotransporting polypeptide (NTCP) was identified as an HBV receptor [9, 10]. Viral entry begins with binding of the externally exposed region of LHBsAg to NTCP [11] . Entry of HBV into uninfected hepatocytes has long been proposed as a potential target for antiviral intervention [12].

In a previous study, we used immortalized human primary hepatocytes, HuS-E/2 cells, as a model to screen for natural, bioactive compounds against HBV infection [13]. We also found that epigallocatechin gallate (EGCG), a flavonoid that belongs to the subclass of catechins and is present in green tea extract, has antiviral and anti-oncogenic properties [14–16] and is able to inhibit HBV entry and contribute to decreased HBV replication in vitro [17]. In this study, we show that EGCG represses the infection of HBV in HuS-E/2 cells.

Fumacrylacetoacetate (Fah) is an enzyme involved in tyrosine metabolism. Fah-deficient mice develop hypoglycemia and liver dysfunction due to the toxicity of accumulated metabolites [18]. In addition, along with the recombination-activating gene2 and IL-2 receptor knock-out (KO) mice that have disrupted immune development, triple KO mice were generated to engraft human cells in a chimeric model. Previously, FRG (Fah^−/−^/ Rag2^−/−^/ IL-2Rγ^/−^) mice have been used to generate chimeras carrying human primary hepatocytes. The FRG mouse is well-established and has been used for research into viral liver-associated diseases, providing a solid platform for human hepatic xenorepopulation [19, 20]. These robust primary hepatocytes have replaced the traditional immortal hepatoma cells or hepatoblasts as a research model, with their viability and differentiation status [20]. Therefore, the human chimeric mouse model of HBV infection is crucial for analyzing EGCG dosage and the timing of treatment. To sum up, our study shows potential to predict prognosis in the clinical setting and the accessibility of the therapeutic effects of EGCG in human patients. This suggests a robust strategy for therapeutic intervention in HBV infection in order to treat the associated liver diseases.

## Methods

### Cell culture

HuS-E/2 [21], and HepG2.2.15 cells (RRID:CVCL_L855), which stably express the HBV genome, were maintained in Dulbecco’s modified Eagle’s medium supplemented with 10% heat-inactivated fetal calf serum and 100 U/ml of penicillin and 100 μg/ml of streptomycin (Gibco). Both HuS-E/2 and HepG2.2.15 cells were cultured at 37 °C in the presence of 5% CO2.

For HBV infection, HuS-E/2 cells were differentiated by incubation with 2% DMSO for 10 days, as described previously [13].

### HBV infection of cell cultures

HBV infection experiments were performed as described previously [13]. Briefly, HBV particles were isolated and concentrated from HepG2.2.15 cells. Differentiated HuS-E/2 cells were incubated for 20 h with purified HBV at a multiplicity of infection (MOI) of 10, in the presence of 0, 10, or 20 μM EGCG or DMSO as control, then the HBV and EGCG were removed and the culture medium was changed every 3 days for 7 days.

### DNA and RNA isolation, reverse transcription and real-time PCR

Total DNA was extracted with a Genomic DNA isolation kit (Nexttec Biotechnologie, Germany). Total RNA was isolated from cultured cells using TRIzol® reagent (Invitrogen). Reverse transcription was performed with the RNA templates, AMV reverse transcriptase (Roche), and oligo-dT primer. The products were subjected to real-time PCR with primer sets of specific genes and SYBR Green PCR Master Mix (Bio-Rad). The primer sets used for HBV core, HBsAg, cccDNA and GAPDH were described previously [13]. The results were analyzed with the iCycler iQ real-time PCR detection system (Bio-Rad). Plasmid p1.3HBcl was prepared at 10-fold dilutions (2*10^4^–2*10^9^ copies/ml) to generate a standard curve in parallel PCR reactions.

### Animals

Eleven 8-week-old female FRG (Fah^−/−^/ Rag2^−/−^/ IL-2Rg^/−^) mice were housed at room temperature with controlled humidity and on a 12 h/12 h light/dark cycle (lights on at 7.00 a.m.) at the Animal Center of the Academia Sinica, Taipei, Taiwan. The weight reached to 25 g in average at 8 weeks old. The food applied was Picolab Rodent Diet 20 (Lab Supply, Inc). Mice were housed in groups using high quality wood pellet hygienic litter bedding (Lignocel HBK 1500–3000, Rettenmaier & Sönne, Germany). Epigallocatechin gallate (EGCE) was injected through the tail vein. Blood was taken from the inferior vena cava during deep anesthesis. The use of animals for this research was approved by the Animal Research Committee of the Academia Sinica and all procedures followed The Guide for the Care and Use of Laboratory Animals (NIH publication, 85–23, revised 1996) and the guidelines of the Animal Welfare Act, Taiwan. On the day of sacrifice, a laparotomy was performed under ketamine and xylazine anesthesia (intramuscular injection of 100 mg/kg body mass and 5 mg/kg body mass, respectively), and whole-blood samples were collected via cardiac puncture.

### Generation of human liver chimeric (Hu-FRG) mice

To generate human liver chimeric (Hu-FRG) mice, cryo-preserved human hepatocytes were purchased from BD Biosciences (San Jose, CA, USA) and CellzDirect/Invitrogen (Durham, NC, USA). FRG (Fah^−/−^/ Rag2^−/−^/ IL-2Rγ^/−^) mice were transplanted as described previously, except for adopting a protocol of gradually decreasing NTBC in the drinking water [19]. One million viable hepatocytes in 200 μl of William’s E medium (Invitrogen Life Technologies, Carlsbad, CA, USA) were injected intrasplenically via a 27-gauge needle. The transplanted mice were given plain water after surgery. To monitor the transplantation rate of human hepatocytes, small amounts of blood were collected monthly from the tail veins of Hu-FRG mice and the serum human albumin (hAlb) levels were determined using a Human Albumin ELISA Quantitation Set (Bethyl Laboratories, Montgomery, TX, USA) according to the manufacturer’s protocol. It takes about 4 months to reach human serum albumin concentration of ≧1 mg/mL. Total eleven mice were transplanted and six Hu-FRG mice with human serum albumin concentrations of ≧1 mg/mL were selected for use in the HBV infection studies.

### HBV infection of Hu-FRG mice

The Hu-FRG mice were subsequently divided randomly into two groups (HBV, *n* = 3, and HBV + EGCG, *n* = 3). (−)-Eepigallocatechin-3-gallate (EGCG) (≥97.0%, HPLC grade) was purchased from Sigma-Aldrich. An inoculum of 5*10^7^ copies of HBV was injected intraperitoneally on day 1. The mice were injected intravenously with EGCG diluted in sterile saline at a concentration of 10 mg/mL (50 μL/10 g body weight). Injections (50 mg/kg) of EGCG were given twice a day on days 1 to 5 (Fig. 3).

### Serological analysis and tissue characterization of Hu-FRG liver chimerism

After HBV infection, mice were sacrificed at week 4 for serological and intrahepatic analyses. Liver specimens were removed during sacrifice and were snap-frozen in liquid nitrogen for further histological and molecular analyses. Serum HBV DNA was quantified by realtime-qPCR at weeks 2 and 4. Serum HBsAg levels were determined by ELISA Quantitation Set (Bio-Rad Laboratories) according to the manufacturer’s protocol. To stain human hepatocytes, cryostat sections of chimeric mouse livers were immunostained with human Fah antibodies (Cell Signaling) and polyclonal rabbit anti-HBcAg (Abcam) to detect HBV core antigen (HBcAg). Briefly, the liver sections were incubated with Fah and HBcAg antibodies at 4 °C overnight, then followed by Alexa488-conjugated goat IgG and Alexa594-conjugated goat IgG at 37 °C for 1 h. Hoechst 33258 (Sigma-Aldrich) was used along with the secondary antibody to detect nucleus. The immunostained samples were detected by Leica DM6000B microscope.

### Statistical analysis

All values are expressed as mean ± SE. Each value is the mean of at least three animals in each group in vivo experiments. Student’s t-test was used for statistical comparison. * indicates that the values are significantly different from the control (*, *p* < 0.05; **, *P* < 0.01; ***, *P* ≤ 0.001.).

## Results

### Inhibitory effect of EGCG on HBV infection

**Fig. 1:**
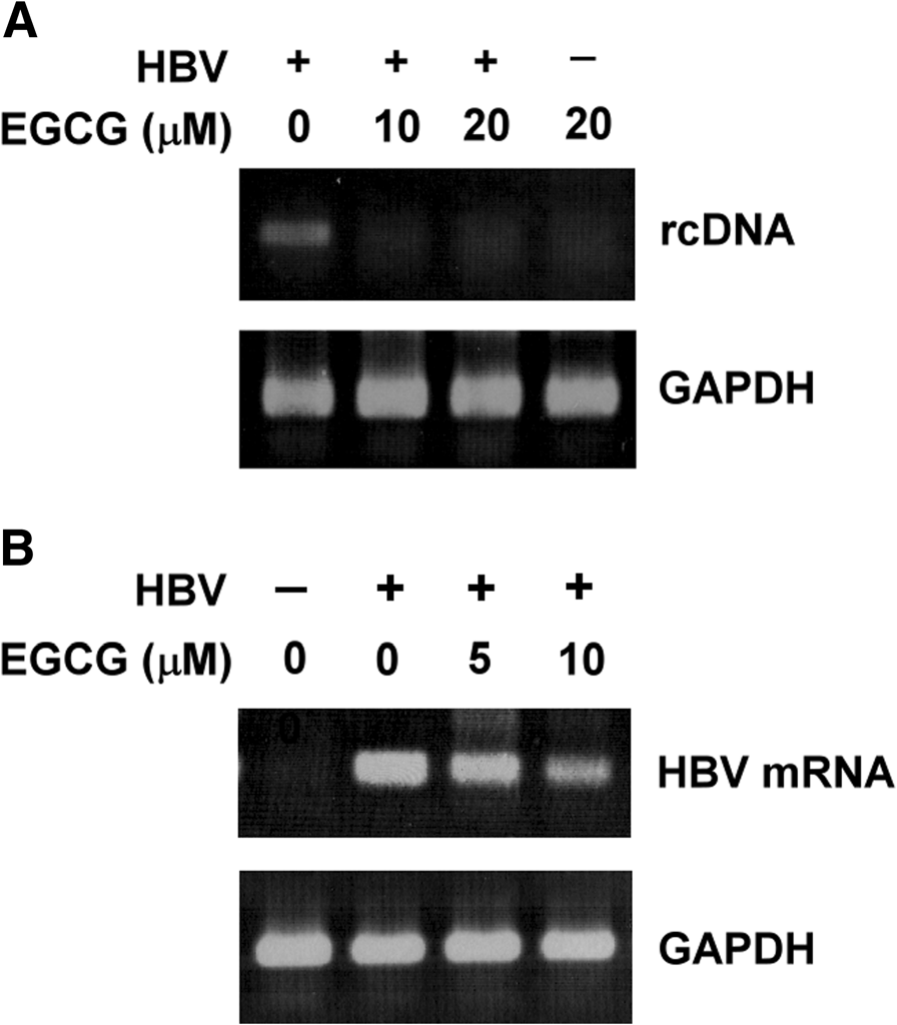
EGCG inhibited HBV infection. DMSO-differentiated HuS-E/2 cells were infected with HBV for 20 h in the presence of EGCG and incubated for an additional 7 days. Nucleic acids were extracted from the cells and amplified to detect the presence of HBV rcDNA (**a**) and HBsAg mRNA (**b**), to evaluate the infection efficiency

To evaluate the effects of EGCG on HBV infectivity and replication, HuS-E/2 cells were infected with HBV derived from HepG2.2.15 cells in the presence of EGCG. The replication efficiency was determined by measuring rcDNA and RNA by PCR and RT-PCR, respectively. EGCG treatment during infection resulted in a dose-dependent decrease of HBV rcDNA (Fig. 1a) and HBsAg mRNA (Fig. 1b) in HuS-E/2 cells. In addition, when the cells were treated with 10 μM EGCG, HBV mRNA levels were reduced by 80% compared to control cells. The half-maximal inhibitory concentration (IC_50_) was estimated to be below 10 μM. Taken together, these results suggest that HBV infection is inhibited by EGCG treatment.

### EGCG inhibited HBV infection in the Hu-FRG mouse model

**Fig. 2:**
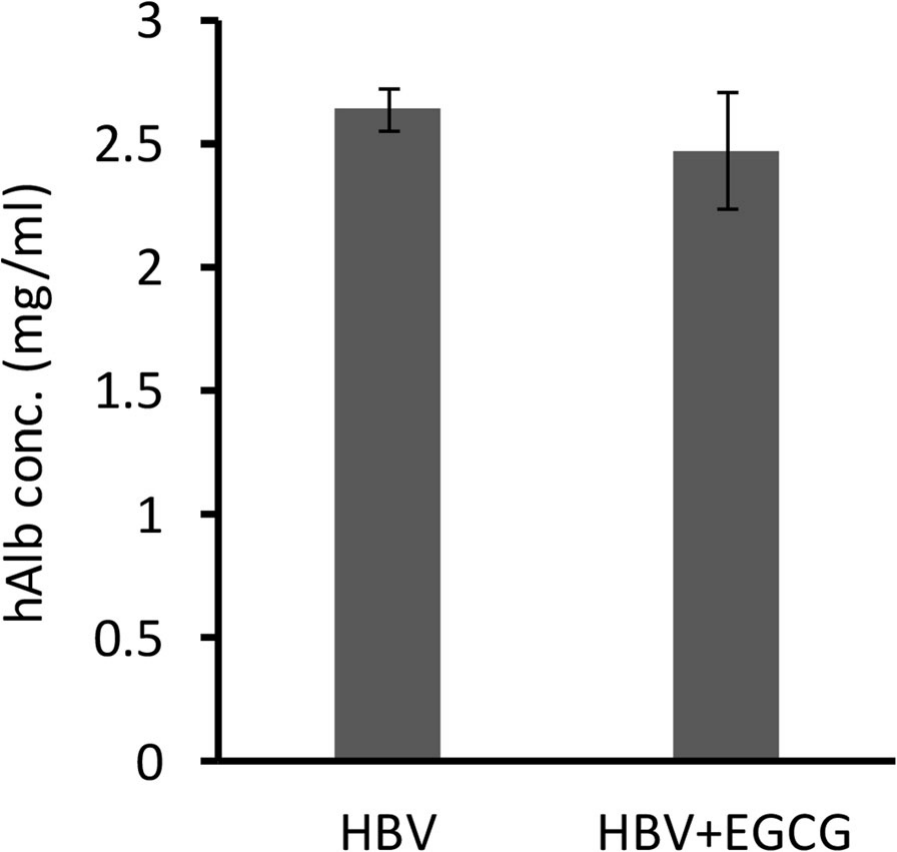
Human albumin levels in Hu-FRG mice. The human albumin level was measured in each mouse. Data were analyzed with mean±SEM and by student t-test (HBV, *n* = 3; HBV + EGCG, *n* = 3)

**Fig. 3:**
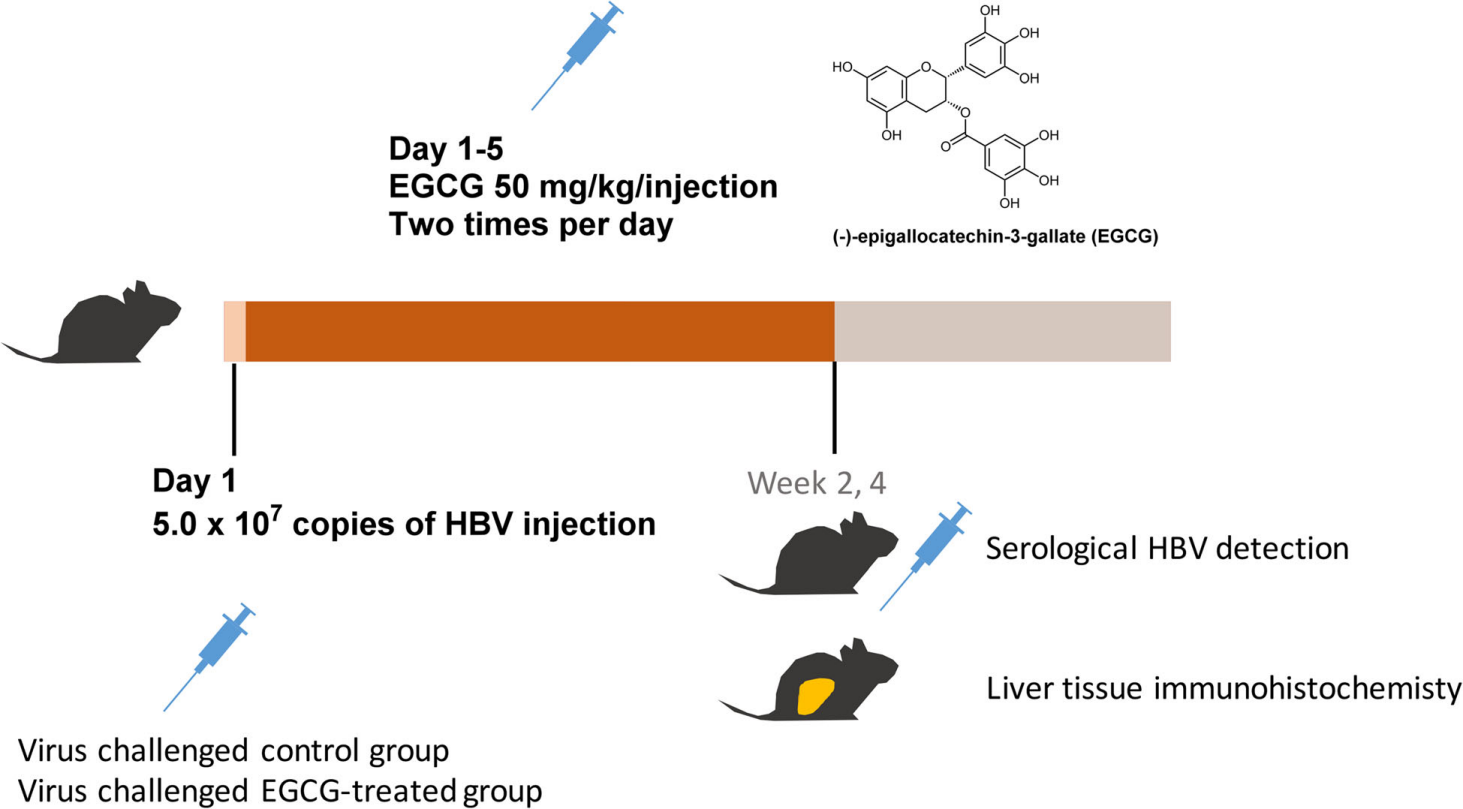
Scheme of EGCG treatment and mouse challenge schedule

**Fig. 4:**
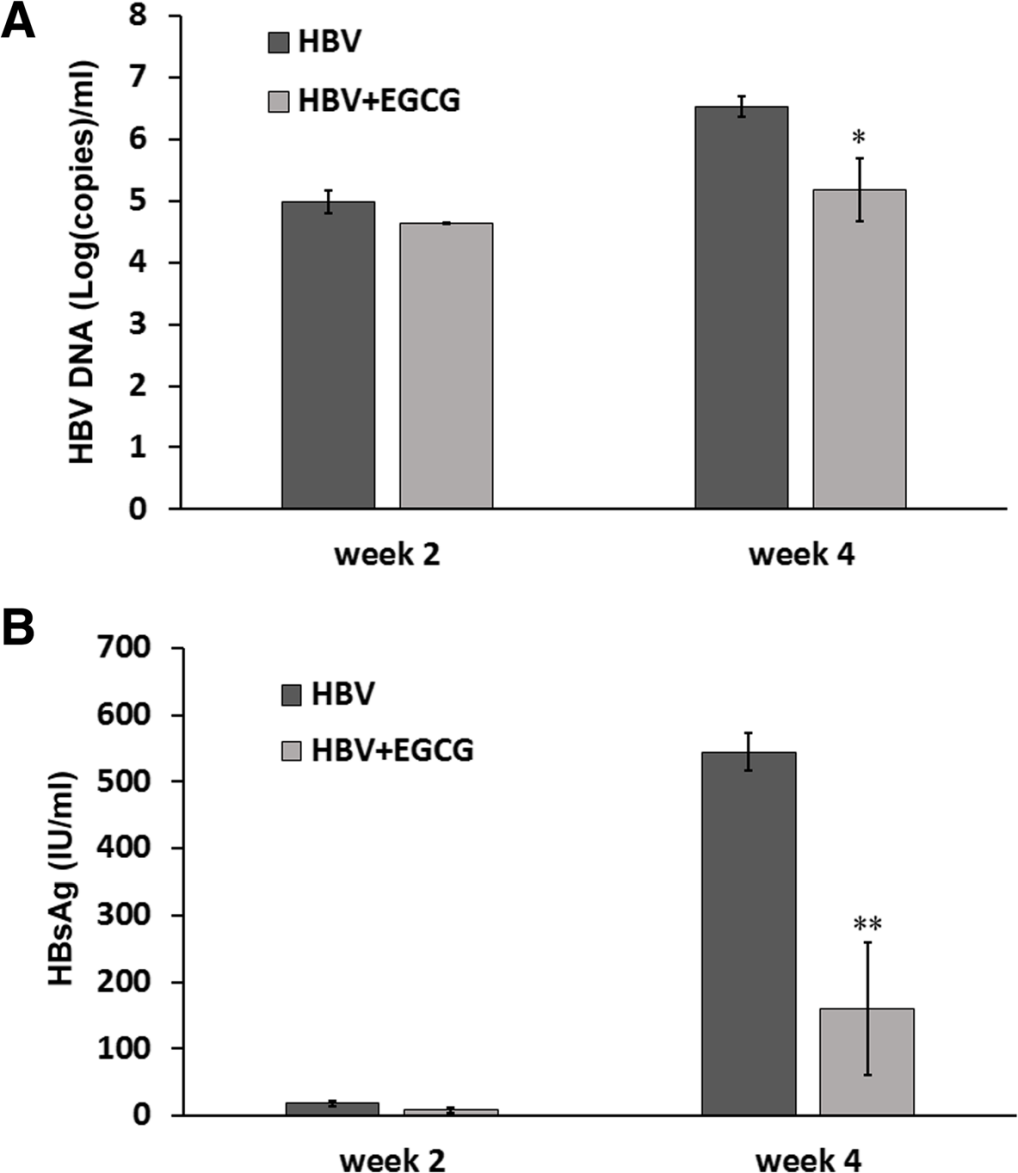
HBV titers in Hu-FRG mice. HBV titers were determined as HBV DNA copies (**a**), and HBV HBsAg expression levels (**b**). The data are expressed with mean±SEM and were analyzed by student t-test (HBV, n=3; HBV+EGCG, n=3; * *p* <0.05, ** *p* <0.01).

To evaluate the EGCG-associated inhibition of HBV infection in vivo, we generated human liver chimeric (Hu-FRG) mice. We measured the human serum albumin (hAlb) concentrations in the Hu-FRG mice and selected mice with ≧1 mg/mL hAlb for the HBV infection studies (Fig. 2). The scheme of EGCG treatment and Hu-FRG mice challenge schedule was shown in Fig. 3. Realtime-qPCR and ELISA assay were used to detect HBV DNA and HBsAg, respectively, in mice serum. Compared to the control group, the HBV DNA copy number was significantly lower (*p* ≤ 0.05) after 4 weeks of treatment with EGCG and challenge with HBV (Fig. 4a). A marked reduction (*p* ≤ 0.01) in HBsAg protein level by 70% was detected (Fig. 4b). These data suggest clearly that EGCG inhibits HBV infection in human liver chimeric mice.

### EGCG inhibited expression of fah and HBcAg in the livers of human chimeric mice

**Fig. 5:**
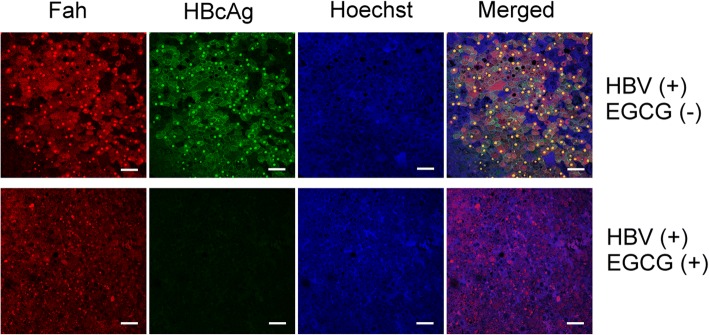
Expression of Fah and HBcAg in the livers of human chimeric mice. Expression of Fah and HBcAg were examined by immunofluorescence staining with antibodies against Fah and HBcAg, followed by confocal microscopy. The bars on the images represent 100 μm

Liver tissue from Hu-FRG chimeric mice was examined to detect HBV infection with/without EGCG treatment. The human Fah-expressing cells were successfully implanted in the mouse livers (Fig. 5) and the HBV infection was monitored by detecting hepatitis B core antigen (HBcAg) expression. We found that, when the mice were treated with EGCG, the levels of expression of HBcAg in the human cells were lower than in the mice without EGCG treatment. Therefore, based on the immunostaining of the liver tissue of the chimeric mice, we confirmed the inhibitory effect of EGCG on HBV infection.

## Discussion

The major components of green tea, polyphenols, which are also known as catechins, have been shown to have a therapeutic effect on a myriad of diseases [22–24]. The catechins in green tea include epicatechin (ECG), epigallocatechin (EGC), epicatechin (EC), catechin (C) and epigallocatechin gallate (EGCG) and EGCG is considered to be the most important in terms of its anti-viral effects through various mechanisms [14, 25]. A previous study by our team showed that EGCG induced clathrin-dependent endocytosis of NTCP and inhibited HBV entry in vitro [17]. In addition, EGCG has been shown to be an inhibitor of the viral serine protease involved in HCV entry [26]. The mechanism of inhibition by EGCG of cellular entry by herpes simplex virus (HSV) may be through disruption of the viral envelope [27]. Several pathways have been reported for the EGCG inhibitory effect on Epstein-Barr virus (EBV) infection, such as interference with the AP-1 pathway [28], reduction of the ability of the virus to bind to host DNA [29], degradation of viral RNA and down-regulation of viral lytic-associated signaling [30].

However, the methods used to elucidate the effects of EGCG were based on cultured cells, including primary human hepatocytes [31]. Therefore, after we confirmed and optimized the EGCG inhibitory efficiency in HuS-E/2 cells, we used a chimeric mouse model to investigate the effect of EGCG on HBV infection in vivo. Previously, an immunodeficient model, based on the urokinase-type plasminogen activator (uPA) mouse, was used for HBV and HCV research [32–34]. To solve the limitation of the uPA mouse model that had high mortality and was difficult to manipulate, the FRG-chimeric mouse model was developed [20]. In our study, we first generated Hu-FRG mice whose livers were repopulated with human hepatocytes. We showed the implantation was efficient by measuring the human albumin level, with no significant difference between the two groups (Fig. 2). Furthermore, we designed an HBV infection strategy and successfully challenged Hu-FRG mice with HBV during EGCG treatment (Fig. 3). Measurement of the HBV DNA copy number and HBsAg titer, as well as immunohistochemical analysis of the liver tissue, provided the solid evidence of inhibition of HBV infection by EGCG (Figs. 4 and 5).

Generally, the HBV DNA copy number exceeded 1.0 × 10^7^ copies/ml 4 weeks after viral challenge in the control group, accompanied by a high titer of HBsAg. We compared the HBV DNA copy number and HBsAg concentration between the untreated and EGCG-treated groups of mice and saw little difference after 2 weeks but, critically, 4 weeks after infection, the HBV titers in the EGCG-treated mice were over 100 fold lower than the control group. To explain the time lag before viral inhibition was apparent, we hypothesized that either the increasing dosage or injection frequency of EGCG blocks HBV entry. We found, despite that the half-life of EGCG is approximately less than 1 h [35], the strategy we applied to boost EGCG was effective and stable enough to block HBV infection. The increasing dosage of EGCG would maintain the EGCG concentration in blood and overcome the degradation of EGCG due to its half-life, which make apparent the effect of EGCG in inhibiting HBV entry into the hepatocytes. Therefore, we may increase the dosage of EGCG in future experiments.

## Conclusions

In the present study, the inhibitory effect of EGCG on HBV infection and replication was demonstrated in vitro. Further, it is clear that the use of the Hu-FRG chimeric mouse model to evaluate the inhibitory effect of EGCG was robust and EGCG may have therapeutic potential for the treatment of HBV-associated liver diseases.

## Abbreviations

EGCG: Epigallocatechin gallate
Fah: Fumacrylacetoacetate
FRG mice: Fah^−/−^/ Rag2^−/−^/ IL-2Rγ^/−^ mice
hAlb: Human albumin
HBcAg: HBV core antigen
HBsAg: Hepatitis B surface antigen
HBV: Hepatitis B virus
NTCP: Sodium taurocholate cotransporting polypeptide
